# A novel age-norm–capped baseline serum creatinine approach for acute kidney injury diagnosis in critically Ill children: a retrospective cohort study

**DOI:** 10.3389/fped.2026.1821197

**Published:** 2026-09-23

**Authors:** Jiaxing Du, Jian Zeng, Jiayu Huang, Xue Lu, Zixuan Jiang, Sujun Chen

**Affiliations:** 1 School of Nursing, Guangzhou Medical University, Guangzhou, Guangdong, China; 2 Pediatric Intensive Care Unit, Dongguan Eighth People’s Hospital (Dongguan Children’s Hospital), Dongguan, Guangdong, China

**Keywords:** acute kidney injury, critically ill children, mortality, Norms-Capped, serum creatinine

## Abstract

**Background:**

A reliable baseline serum creatinine (SCr) for pediatric acute kidney injury (AKI) diagnosis is often missing, and simply using admission SCr may underestimate AKI. We aimed to evaluate a novel approach for defining baseline SCr by comparing its implications for AKI classification and prognosis with those of admission SCr.

**Methods:**

This retrospective study utilized data from the Paediatric Intensive Care (PIC) database. We compared two distinct baseline SCr definitions: 1) the “Admission SCr” and 2) a novel “Norms-Capped SCr”, defined as the admission SCr if it was less than or equal to the age-specific normal maximum (NormsMax), or the NormsMax value if the admission SCr exceeded it. Both baseline definitions were then used to classify AKI according to three different diagnostic criteria. We subsequently compared the resulting AKI incidence, stage distribution, and prognostic performance for clinical outcomes across these different baseline-criterion combinations.

**Results:**

A total of 3,021 critically ill children were included. Norms-Capped SCr identified more AKI than admission SCr across KDIGO (41.7% vs. 27.4%), pROCK (38.7% vs. 27.5%), and pRIFLE (55.1% vs. 32.6%). It also showed more consistent associations with in-hospital mortality, although pRIFLE Stage 3 was not significant. Apparent AUCs ranged from 0.797 to 0.817 for Norms-Capped SCr models and from 0.783 to 0.785 for admission SCr models; the corresponding optimism-corrected AUCs were 0.784–0.804 and 0.767–0.770, respectively. Calibration was comparable, while prediction error was slightly lower with Norms-Capped SCr.

**Conclusion:**

The Norms-Capped SCr approach showed modestly better overall performance than admission SCr for defining baseline SCr in critically ill children without known pre-admission values, particularly when combined with the pROCK criteria.

## Introduction

Acute kidney injury (AKI) is a frequent and serious complication in critically ill children, affecting approximately 25%–50% of pediatric intensive care unit (PICU) admissions (1–3), with 10%–12% of patients developing severe (Stage 2–3) disease (4, 5). The immediate consequences of AKI are severe: it is independently associated with prolonged mechanical ventilation, longer ICU and hospital stays, and a near-doubling of mortality risk (6–10). The long-term impact is also profound, as approximately 10%–18% of children surviving an AKI episode subsequently develop chronic kidney disease (11, 12). Given these adverse outcomes, the accurate and timely diagnosis of AKI is a paramount priority in pediatric critical care.

The accurate diagnosis of AKI is critically dependent on comparing a patient's current serum creatinine (SCr) to a reliable baseline (13, 14). However, this presents a major challenge in PICUs, where a true, pre-illness steady-state SCr is rarely available (15, 16). To overcome this, clinicians and researchers often use the admission SCr (AdmSCr) as the surrogate baseline. This common practice is flawed. A significant proportion of critically ill children already have community-acquired AKI or dehydration upon arrival, meaning their admission SCr is already elevated (17, 18). This inaccuracy obscures the true association between AKI and adverse outcomes, impeding risk stratification and delaying interventions (19), which highlights an urgent need for a practical alternative to address the potential under-classification of AKI associated with the AdmSCr method.

To address this, we propose and formally evaluate a novel “Norms-Capped SCr” baseline, defined as the lesser of either the patient's admission SCr or the age-specific upper limit of normal (NormsMax) (20). The rationale is to prevent the common error of “normalizing” an already elevated admission SCr; by capping the baseline at the highest expected physiologic value, any renal dysfunction present on admission is captured rather than masked. We hypothesize that this Norms-Capped SCr method will classify a greater proportion of patients as having AKI than the AdmSCr method and, as suggested by prior analyses, offer superior prognostic discrimination for adverse outcomes.

Therefore, our primary objective is to compare the Norms-Capped SCr and AdmSCr baseline methods by assessing their impact on AKI classification and prognostic performance across the Kidney Disease: Improving Global Outcomes (KDIGO), Pediatric Reference Change Value Optimized for AKI (pROCK), and Pediatric Risk, Injury, Failure, Loss, End-Stage Renal Disease (pRIFLE) criteria. As the comparative superiority of the KDIGO, pROCK, and pRIFLE criteria remains unclear (21, 22), our secondary objective is to compare their prognostic performance within this cohort.

## Methods

### Study design and data source

We conducted a retrospective cohort study using data from the Paediatric Intensive Care (PIC) database, encompassing admissions from 2010 to 2018. The PIC database is a large clinical registry containing de-identified data, including laboratory results and clinical outcomes (23). For this analysis, we included all critically ill children aged 28 days to 18 years. The establishment and use of the database for research were approved by the Institutional Review Board of the Children's Hospital, Zhejiang University School of Medicine. Jiaxing Du obtained the required data-use permission and signed a data use agreement before accessing the database.

### Patient selection

Baseline characteristics were defined as demographic and laboratory variables recorded within the first 24 h after PICU admission. Patients were excluded if they had fewer than two SCr measurements during the PICU stay or had missing data for the outcome or covariates included in the final models. Patients with documented chronic kidney disease (CKD) were also excluded. Other pre-existing renal diseases were not separately excluded unless they were documented as chronic kidney disease. The extent of missingness for each variable was summarized, and the characteristics of included and excluded patients were compared. The primary analyses were conducted using complete cases. The detailed participant-selection process is presented in Supplementary Figure S1.

### Definitions of baseline SCr and AKI

Two approaches were used to define baseline SCr: (1) admission SCr, defined as the first SCr measurement obtained at PICU admission; and (2) Norms-Capped SCr, defined as the lower of admission SCr and the age-specific upper reference limit for SCr (NormsMax). The age-specific NormsMax values and their sources are provided in Supplementary Table S1.

AKI occurrence and stage were assessed separately using the KDIGO, pROCK, and pRIFLE criteria under each baseline SCr approach. Because urine output data were unavailable, AKI identification using the KDIGO and pRIFLE criteria was based exclusively on their serum creatinine components. All SCr measurements obtained from ICU admission to discharge were considered. Peak SCr was defined as the highest SCr value recorded during this period, and the highest AKI stage reached during the same period was recorded as the final AKI stage. The detailed definitions and staging thresholds for the three AKI criteria are provided in Supplementary Table S2.

### Covariates

Covariates were selected *a priori* based on clinical relevance, evidence from previous studies (3, 9), availability at baseline, and their potential roles as confounders. The continuous covariates included age, chloride, hemoglobin, glucose, neutrophil, partial pressure of carbon dioxide, platelet count, and albumin. Sex and ICU types were included as categorical covariates. All laboratory covariates were defined using the first available measurement within 24 h of PICU admission.

### Statistical analysis

Continuous variables were summarized as medians with interquartile ranges, and categorical variables were summarized as counts and percentages. The incidence and stage distribution of AKI were calculated for each combination of AKI criterion and baseline SCr approach. The primary outcome was in-hospital mortality, defined as death occurring during hospitalization. Patients without an in-hospital death were classified as non-events.

Multivariable logistic regression models were used to examine the associations of AKI stage, any AKI, and severe AKI with in-hospital mortality, with each AKI representation evaluated in a separate model. Results are reported as adjusted odds ratios (ORs) with 95% confidence intervals (CIs). All models were adjusted for the prespecified covariates described in the Covariates section.

Before model fitting, multicollinearity was assessed using pairwise correlation coefficients among continuous covariates and variance inflation factors (VIFs) for the fitted models. Model complexity was assessed using events per predictor parameter. Internal validation was performed using 1,000 bootstrap resamples. In each resample, the model was refitted and optimism was estimated as the difference between performance in the bootstrap sample and the original dataset. Mean optimism was subtracted from the apparent performance, and approximate 95% CIs were obtained from the 2.5th and 97.5th percentiles of the distribution of apparent performance minus bootstrap-specific optimism.

Discrimination was evaluated using ROC curves and AUCs derived from the predicted probabilities of the full multivariable logistic regression models. For each AKI criterion and representation, the apparent AUCs of the corresponding Norms-Capped SCr and admission SCr models were formally compared using paired bootstrap resampling. The difference in AUC (ΔAUC) was calculated as AUC (Norms-Capped)—AUC (Admission), with 95% CIs and *P* values obtained from 1,000 paired bootstrap resamples.

Calibration was assessed using the calibration intercept and slope, and overall prediction error was evaluated using the Brier score. Because apparent calibration intercepts of approximately 0 and slopes of approximately 1 are expected when models are evaluated in the same data used for model development, interpretation focused on the optimism-corrected calibration estimates. Threshold-based performance was assessed at cutoffs selected using the Youden index, including sensitivity, specificity, positive predictive value, negative predictive value, and accuracy with exact binomial 95% CIs. These threshold-based analyses were considered exploratory because the cutoffs were selected and evaluated in the same dataset. All statistical tests were two-sided, and *P* < 0.05 was considered statistically significant. Analyses were performed using R software, version 4.5.1.

## Results

A total of 3,021 critically ill children were included in the final analysis. The cohort had a median age of 1.33 years (IQR 0.42–4.08), included 1,684 (55.7%) males, and experienced an overall in-hospital mortality rate of 7.1% (*n* = 214). Detailed baseline characteristics stratified by in-hospital mortality are presented in Table 1. The extent of missingness for each variable is reported in Supplementary Table S3. Baseline characteristics of included and excluded patients were compared using standardized mean differences and are presented in Supplementary Table S4. The baseline SCr definition substantially affected the detected incidence of AKI. Compared with admission SCr, Norms-Capped SCr identified a higher incidence of AKI across all three criteria: KDIGO (41.7% vs. 27.4%), pROCK (38.7% vs. 27.5%), and pRIFLE (55.1% vs. 32.6%). The detailed distributions of AKI stages for each combination of AKI criterion and baseline SCr approach are shown in Figure 1.

**Table 1:** Baseline characteristics overall and stratified by survival status.

Characteristic	Overall	Survivors	Non-survivors	*p*-value
Age, years	1.33 [0.42, 4.08]	1.33 [0.42, 4.08]	1.08 [0.25, 3.90]	0.089
Sex, *n* (%)				0.017
Male	1,684 (55.7%)	1,548 (55.1%)	136 (63.6%)	
Female	1,337 (44.3%)	1,259 (44.9%)	78 (36.4%)	
Hemoglobin, g/L	105 [93, 118]	105 [93, 118]	108 [89, 127]	0.281
Glucose, mmol/L	7.8 [5.9, 10.8]	7.9 [6.0, 10.8]	6.1 [5.0, 10.8]	<0.001
Neutrophil, %	68.8 [55.9, 79.4]	69.1 [56.6, 79.4]	61.7 [43.7, 78.4]	<0.001
Platelet, 10^9^/L	209 [141, 314]	209 [142, 312]	228 [109, 356]	0.603
PaCO2, mmHg	37.4 [32.3, 43.6]	37.4 [32.4, 43.5]	38.1 [31.9, 46.4]	0.309
Chloride, mmol/L	108 [104, 111]	108 [105, 111]	106 [102, 110]	<0.001
Albumin, g/L	38.50 [34.70, 41.80]	38.60 [35.00, 41.95]	35.30 [30.40, 40.08]	<0.001
ICU types, *n* (%)				<0.001
CICU	1,341 (44.4%)	1,319 (47.0%)	22 (10.3%)	
PICU	443 (14.7%)	367 (13.1%)	76 (35.5%)	
SICU	792 (26.2%)	704 (25.1%)	88 (41.1%)	
General ICU	445 (14.7%)	417 (14.9%)	28 (13.1%)	

**Figure 1:**
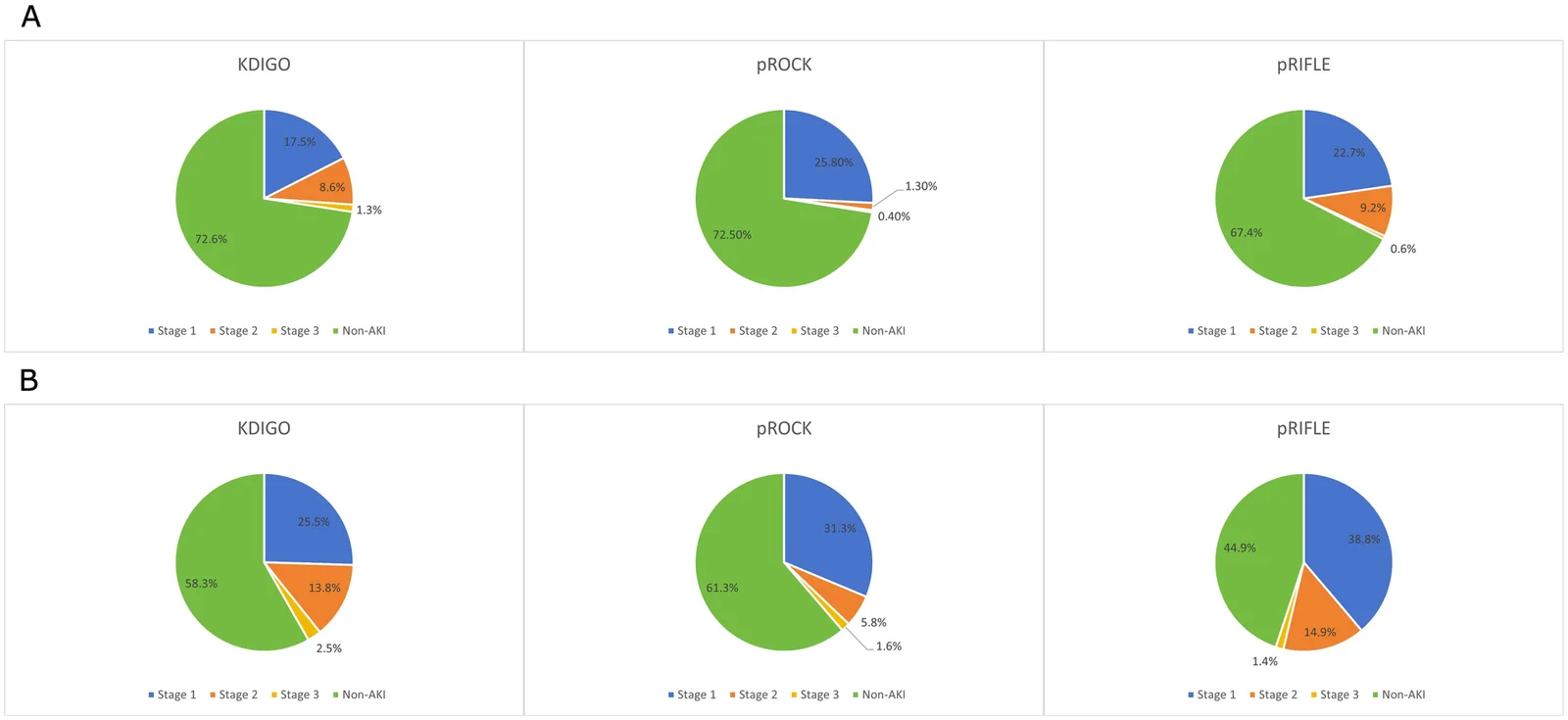
Impact of different baseline SCr estimation methods on the incidence and staging of AKI. **(A)** AdmSCr and **(B)** Norms-Capped SCr. The charts compare AKI incidence and staging by KDIGO, pROCK, and pRIFLE criteria.

After adjustment for the prespecified baseline covariates, AKI defined using Norms-Capped SCr showed more consistent associations with in-hospital mortality than AKI defined using admission SCr (Figure 2). Under the Norms-Capped approach, all KDIGO and pROCK stages were significantly associated with in-hospital mortality, with the strongest association observed for pROCK Stage 3 (OR 7.66, 95% CI 3.93–14.93). For pRIFLE, Stages 1 and 2 were associated with in-hospital mortality, whereas Stage 3 was not (OR 1.34, 95% CI 0.48–3.72; *P* = 0.574). Any AKI and severe AKI were associated with in-hospital mortality across all three criteria. In contrast, under the admission SCr approach, only KDIGO Stage 1 and pROCK-defined severe AKI were significantly associated with in-hospital mortality, while the remaining stage-specific, any-AKI, and severe-AKI estimates were not statistically significant.

**Figure 2:**
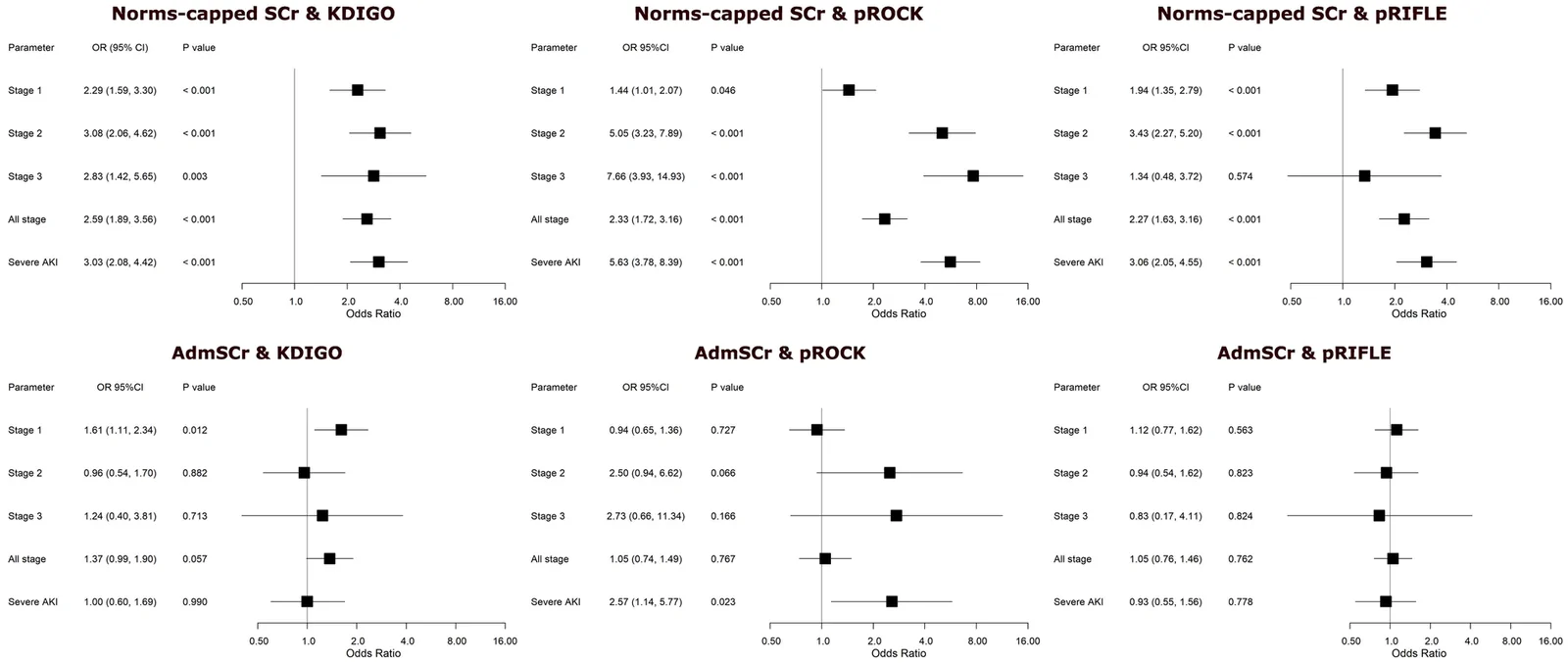
Impact of different baseline SCr estimation methods on the association between AKI and mortality. All models were adjusted for age, sex, ICU types, chloride, hemoglobin, glucose, neutrophil, platelet, PaCO_2_ and albumin. ICU, intensive care unit.

The final models had events per predictor parameter (EPP) values ranging from 14.27 to 16.46. For the stage-based models, the apparent AUCs ranged from 0.801 to 0.817 for Norms-Capped SCr and from 0.784 to 0.785 for AdmSCr (Figure 3A). For the any-AKI models, the corresponding AUCs ranged from 0.797 to 0.802 and from 0.783 to 0.784, respectively (Figure 3B). For the severe-AKI models, the AUCs ranged from 0.800 to 0.817 for Norms-Capped SCr and from 0.784 to 0.785 for AdmSCr (Figure 3C). The highest apparent AUC was observed for the Norms-Capped SCr model incorporating pROCK stage or severe AKI (AUC 0.817). After bootstrap internal validation, the optimism-corrected AUCs ranged from 0.784 to 0.804 for the Norms-Capped SCr models and from 0.767 to 0.770 for the AdmSCr models (Table 2). Paired bootstrap comparisons showed significantly higher apparent AUCs for the Norms-Capped SCr models across all nine comparisons (Supplementary Table S5).

**Figure 3:**
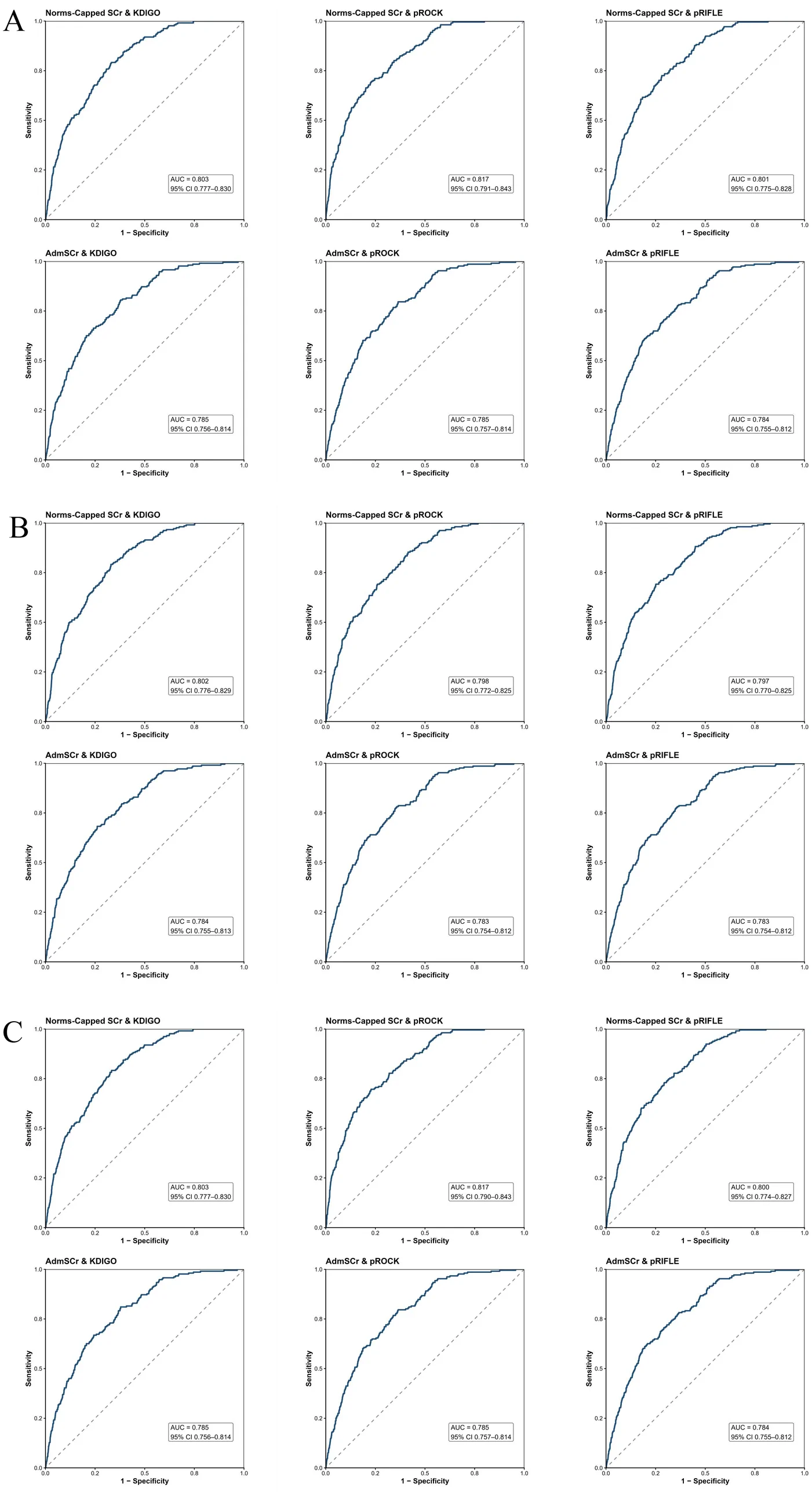
ROC curves for predicting mortality using different baseline SCr approaches. **(A)** AKI stage; **(B)** any AKI; and **(C)** severe AKI.

**Table 2:** Apparent and bootstrap optimism-corrected performance of multivariable logistic regression models for mortality.

Baseline SCr method	AKI form	EPP	AUC (95% CI)	Corrected AUC (95% CI)	Calibration intercept (95% CI)	Corrected intercept (95% CI)	Calibration slope (95% CI)	Corrected slope (95% CI)	Brier score	Corrected Brier score (95% CI)
AdmSCr & KDIGO	AKI Stage	14.27	0.785 (0.756, 0.814)	0.769 (0.739, 0.800)	0.000 (−0.146, 0.146)	0.006 (−0.132, 0.145)	1.000 (0.848, 1.152)	1.000 (0.836, 1.141)	0.060	0.061 (0.055, 0.068)
	Any AKI	16.46	0.784 (0.755, 0.813)	0.770 (0.739, 0.799)	0.000 (−0.146, 0.146)	0.006 (−0.132, 0.146)	1.000 (0.847, 1.153)	1.000 (0.840, 1.138)	0.060	0.061 (0.055, 0.068)
	Severe AKI	15.28	0.785 (0.756, 0.814)	0.770 (0.740, 0.801)	0.000 (−0.146, 0.146)	0.005 (−0.133, 0.146)	1.000 (0.848, 1.152)	1.000 (0.838, 1.140)	0.060	0.061 (0.055, 0.068)
Norms-Capped SCr & KDIGO	AKI Stage	14.27	0.803 (0.777, 0.830)	0.789 (0.762, 0.817)	0.000 (−0.148, 0.148)	0.005 (−0.137, 0.150)	1.000 (0.858, 1.142)	0.999 (0.851, 1.121)	0.059	0.061 (0.054, 0.068)
	Any AKI	16.46	0.802 (0.776, 0.829)	0.790 (0.762, 0.818)	0.000 (−0.148, 0.148)	0.005 (−0.137, 0.149)	1.000 (0.857, 1.143)	0.999 (0.852, 1.125)	0.059	0.061 (0.054, 0.067)
	Severe AKI	15.28	0.803 (0.777, 0.830)	0.790 (0.763, 0.817)	0.000 (−0.148, 0.148)	0.005 (−0.138, 0.150)	1.000 (0.858, 1.142)	0.999 (0.850, 1.121)	0.059	0.061 (0.054, 0.067)
AdmSCr & pRIFLE	AKI Stage	14.27	0.784 (0.755, 0.812)	0.767 (0.737, 0.798)	0.000 (−0.146, 0.146)	0.006 (−0.132, 0.145)	1.000 (0.846, 1.154)	1.001 (0.841, 1.139)	0.060	0.062 (0.055, 0.068)
	Any AKI	16.46	0.783 (0.754, 0.812)	0.769 (0.740, 0.799)	0.000 (−0.146, 0.146)	0.006 (−0.132, 0.145)	1.000 (0.846, 1.154)	1.001 (0.840, 1.140)	0.060	0.061 (0.055, 0.068)
	Severe AKI	15.28	0.784 (0.755, 0.812)	0.769 (0.740, 0.799)	0.000 (−0.146, 0.146)	0.006 (−0.132, 0.145)	1.000 (0.846, 1.154)	1.001 (0.839, 1.140)	0.060	0.062 (0.055, 0.068)
Norms-Capped SCr & pRIFLE	AKI Stage	14.27	0.801 (0.775, 0.828)	0.787 (0.759, 0.814)	0.000 (−0.148, 0.148)	0.005 (−0.135, 0.149)	1.000 (0.857, 1.143)	0.999 (0.859, 1.128)	0.059	0.061 (0.054, 0.067)
	Any AKI	16.46	0.797 (0.770, 0.825)	0.784 (0.756, 0.814)	0.000 (−0.147, 0.147)	0.005 (−0.133, 0.150)	1.000 (0.854, 1.146)	0.999 (0.848, 1.127)	0.06	0.061 (0.055, 0.068)
	Severe AKI	15.28	0.800 (0.774, 0.827)	0.787 (0.759, 0.815)	0.000 (−0.147, 0.147)	0.005 (−0.137, 0.152)	1.000 (0.856, 1.144)	0.999 (0.854, 1.125)	0.059	0.061 (0.054, 0.068)
AdmSCr & pROCK	AKI Stage	14.27	0.785 (0.757, 0.814)	0.769 (0.739, 0.798)	0.000 (−0.146, 0.146)	0.005 (−0.134, 0.146)	1.000 (0.848, 1.152)	1.000 (0.842, 1.140)	0.060	0.062 (0.055, 0.068)
	Any AKI	16.46	0.783 (0.754, 0.812)	0.769 (0.739, 0.799)	0.000 (−0.146, 0.146)	0.006 (−0.132, 0.145)	1.000 (0.846, 1.154)	1.001 (0.841, 1.140)	0.060	0.061 (0.055, 0.068)
	Severe AKI	15.28	0.785 (0.757, 0.814)	0.770 (0.740, 0.799)	0.000 (−0.146, 0.146)	0.005 (−0.134, 0.146)	1.000 (0.848, 1.152)	1.000 (0.841, 1.139)	0.060	0.061 (0.055, 0.068)
Norms-Capped SCr & pROCK	AKI Stage	14.27	0.817 (0.791, 0.843)	0.804 (0.778, 0.830)	0.000 (−0.150, 0.150)	0.005 (−0.142, 0.155)	1.000 (0.869, 1.131)	0.999 (0.868, 1.114)	0.058	0.060 (0.053, 0.066)
	Any AKI	16.46	0.798 (0.772, 0.825)	0.786 (0.759, 0.815)	0.000 (−0.147, 0.147)	0.005 (−0.133, 0.150)	1.000 (0.855, 1.145)	1.000 (0.850, 1.129)	0.059	0.061 (0.054, 0.067)
	Severe AKI	15.28	0.817 (0.790, 0.843)	0.804 (0.777, 0.830)	0.000 (−0.150, 0.150)	0.005 (−0.140, 0.155)	1.000 (0.869, 1.131)	0.999 (0.867, 1.117)	0.058	0.059 (0.053, 0.066)

AUCs were calculated from the predicted probabilities of the full multivariable logistic regression models. Optimism-corrected estimates were obtained using 1,000 bootstrap resamples.

The optimism-corrected calibration intercepts ranged from 0.005 to 0.006, and the corresponding calibration slopes ranged from 0.999 to 1.001 across all models. The calibration plots are presented in Supplementary Figure S2. Apparent Brier scores ranged from 0.058 to 0.060, while optimism-corrected Brier scores ranged from 0.059 to 0.062. The lowest corrected Brier score was observed for the Norms-Capped SCr model incorporating pROCK-defined severe AKI. At the optimized probability thresholds, sensitivity ranged from 0.626 to 0.853, specificity from 0.585 to 0.791, PPV from 0.134 to 0.186, NPV from 0.966 to 0.982, and accuracy from 0.604 to 0.780. Detailed threshold-based performance estimates with 95% confidence intervals are presented in Supplementary Table S6.

## Discussion

The accurate diagnosis and staging of AKI in children rely heavily on accurate determination of baseline SCr (24). However, establishing a reliable baseline SCr value in clinical practice, particularly in the PICU setting, remains challenging (25). Currently, methods employed in both research and clinical settings to estimate or determine pediatric baseline SCr primarily include: (1) AdmSCr methods using the admission SCr value directly as the baseline (26); (2) Modified AdmSCr method: selecting the lowest SCr value measured during hospitalization (within the first 7 or 14 days) if AdmSCr ≥ 1.2 mg/dL (27, 28); (3) using the average of multiple SCr results from previous outpatient follow-ups during a stable period (1–12 months prior to admission) (19, 29); and (4) Schwartz method: back-calculating the baseline SCr using the Schwartz formula based on an assumed ideal estimated glomerular filtration rate (eGFR) (30). Despite the availability of these methods, no consensus has been reached on a “gold standard” definition.

To address this challenge, we evaluated an alternative approach for defining baseline SCr in the PICU, termed the Norms-Capped SCr. This approach is founded on two practical considerations. First, in the common clinical scenario where critically ill children lack a true pre-admission SCr history, the AdmSCr remains the most immediate and universally available data point (31). However, the second consideration is that, for patients without CKD, relying solely on AdmSCr is inherently flawed and risks significant underestimation of AKI (32). This diagnostic “blind spot” becomes more perilous in the very patients who are most severely ill. For instance, based on pROCK criteria, a patient with a true baseline of 0.4 mg/dL presenting with an AdmSCr of 1.0 mg/dL is already in AKI Stage 3. If this elevated AdmSCr is incorrectly used as the baseline, the existing severe AKI is completely masked, and the diagnostic threshold for subsequent injury is significantly and artificially raised.

While the application of alternatives like the Modified AdmSCr method and the eGFR-based Schwartz method has, to some extent, compensated for the shortcomings of the AdmSCr-only approach, they each introduce their own new and significant limitations. The modified AdmSCr method can only be applied retrospectively because the nadir SCr depends on subsequent measurements and is therefore unavailable at the time of initial AKI assessment. It may also be less reliable in patients with persistent AKI or early mortality, in whom the observed nadir may remain elevated (33–35). Schwartz-based back-calculation assumes a normal eGFR and may not fully account for inter-individual variability. It may produce a lower estimated baseline SCr and, as noted by Holmes et al., increase the risk of AKI overdiagnosis or over-reporting (36).

To meet this need, our Norms-Capped SCr method applies a critical “insurance policy” by integrating the Normax as a ceiling. We acknowledge that some studies have cautioned that using Normax universally for all patients might also lead to inaccuracies, particularly in children with a very low true baseline (13, 37). We posit that when an AdmSCr is already abnormally high (AdmSCr > Normax), it is a strong signal of pre-existing injury, and using this elevated value as the reference point is clinically unsound. By establishing Normax as the ceiling in these specific instances, we create a more plausible “healthy” baseline, thereby rescuing the diagnosis for the most vulnerable patients.

Our results suggest that models incorporating Norms-Capped SCr showed modestly better discrimination and slightly lower overall prediction error than the corresponding models incorporating AdmSCr. However, the relatively low PPVs (0.134–0.186) limit the utility of these models as stand-alone tools for identifying individual patients at high risk of mortality. Their clinical value should be interpreted primarily in the context of risk stratification. Norms-Capped SCr also identified a higher proportion of patients as having AKI and reclassified 11.2%–22.5% of the cohort from non-AKI to AKI. However, in the absence of an independent reference standard, it cannot be determined whether these additional classifications represented more accurate identification of AKI. The inclusion of these additionally identified patients may partly explain the more consistent associations between AKI categories and in-hospital mortality under the Norms-Capped approach. Notably, pRIFLE Stage 3 was not significantly associated with in-hospital mortality when either Norms-Capped SCr (OR 1.34, 95% CI 0.48–3.72; *P* = 0.574) or AdmSCr (OR 0.83, 95% CI 0.17–4.11; *P* = 0.824) was used, indicating that this finding was not specific to the Norms-Capped approach. The wide confidence intervals under both baseline definitions suggest limited precision, potentially because of the small number of patients or in-hospital mortality events in this category.

The second objective of this study was to compare the prognostic performance of the three AKI criteria. Based on the overall findings, pROCK showed the clearest stage-dependent association with in-hospital mortality under the Norms-Capped SCr approach. The adjusted ORs increased from 1.44 (95% CI 1.01–2.07) for Stage 1 to 5.05 (95% CI 3.23–7.89) for Stage 2 and 7.66 (95% CI 3.93–14.93) for Stage 3. The full multivariable model incorporating pROCK stage also showed the highest apparent AUC (0.817), although differences in AUC among the three criteria were modest. Previous studies have likewise reported associations between pROCK-defined AKI and adverse outcomes in children, including adverse kidney outcomes, complications, and mortality (21, 38).

This study has several limitations. Its single-center, retrospective design may limit generalizability, and the exclusion of patients with fewer than two SCr measurements or missing covariates may have introduced selection bias. Data on some clinical interventions and long-term renal outcomes were unavailable. Missing height and prior outpatient SCr data precluded comparison with other baseline SCr approaches, while inpatient nadir SCr was not evaluated because it may be influenced by the clinical course and treatment and may not reliably represent pre-illness kidney function. Furthermore, because AKI severity was defined using the highest stage reached during hospitalization, the mortality analyses reflect associations with maximum AKI severity and should not be interpreted as admission-time or real-time prognostic performance. The applicability of CALIPER-derived NormsMax values to critically ill Chinese children may be limited. In addition, KDIGO and pRIFLE classifications were based on serum creatinine alone because urine output data were unavailable. Although bootstrap internal validation was performed, external validation is required.

## Conclusion

In critically ill children without known pre-admission SCr values, the Norms-Capped SCr approach may provide a practical alternative for defining baseline SCr. Compared with admission SCr, it showed more consistent AKI risk stratification and modestly better overall model performance, with the most favorable results observed when combined with the pROCK criteria. Further external validation in independent pediatric cohorts is required before clinical application.

## Data Availability

The raw data supporting the conclusions of this article will be made available by the authors, without undue reservation.
