# Supplementary material for: A novel age-norm–capped baseline serum creatinine approach for acute kidney injury diagnosis in critically Ill children: a retrospective cohort study
Source: Front Pediatr. 2026 Sep 23;14:1821197. doi: 10.3389/fped.2026.1821197 (PMC13651550; doi:10.3389/fped.2026.1821197)
Supplement: Supplementary file 1 [file Supplementaryfile1.docx]

**Supplementary**

**eTable 1.** Age- and sex-specific pediatric reference intervals for serum creatinine

**eTable 2.** AKI staging criteria (pRIFLE, KDIGO, and pROCK) based on serum creatinine.

**eTable 3.** Extent of variable-specific missing data among otherwise eligible patients.

**eTable 4.** Comparison of baseline characteristics between included and excluded patients.

**eTable 5.** Paired AUC comparison of Norms-Capped and admission SCr models.

**eTable 6.** Threshold-based classification performance of multivariable logistic regression models for mortality.

**eFigure 1.** Diagram of cases included in the study and AKI incidence.

**eFigure 2.** Calibration plots for predicting mortality using different baseline SCr approaches.

**eTable 1.** Age- and sex-specific pediatric reference intervals for serum creatinine.

| **Analyte** | **Age** | **Female** | | **Male** | |
| --- | --- | --- | --- | --- | --- |
|  |  | **Upper limit (NormsMax)** | **Upper-limit 95% CI** | **Upper limit (NormsMax)** | **Upper-limit 95% CI** |
| Creatinine (enzymatic), mg/dL | 0 to 14 days | **0.92** | 0.90–0.98 | **0.92** | 0.90–0.98 |
| Creatinine (enzymatic), mg/dL | 15 days to <2 years | **0.36** | 0.35–0.38 | **0.36** | 0.35–0.38 |
| Creatinine (enzymatic), mg/dL | 2 to <5 years | **0.43** | 0.41–0.45 | **0.43** | 0.41–0.45 |
| Creatinine (enzymatic), mg/dL | 5 to <12 years | **0.61** | 0.60–0.62 | **0.61** | 0.60–0.62 |
| Creatinine (enzymatic), mg/dL | 12 to <15 years | **0.81** | 0.76–0.85 | **0.81** | 0.76–0.85 |
| Creatinine (enzymatic), mg/dL | 15 to <19 years | **0.84** | 0.81–0.88 | **1.08** | 1.06–1.11 |

NormsMax denotes the upper reference limit for serum creatinine.

Reference source: Colantonio DA, Kyriakopoulou L, Chan MK, et al. Closing the Gaps in Pediatric Laboratory Reference Intervals: A CALIPER Database of 40 Biochemical Markers in a Healthy and Multiethnic Population of Children. Clin Chem. 2012;58(5):854–868.

**eTable 2.** AKI staging criteria (pRIFLE, KDIGO, and pROCK) based on serum creatinine.

| Stages**^a^** | **2007 pRIFLE*** | **2012 KDIGO** | **2018 pROCK** |
| --- | --- | --- | --- |
| Stage 1 | 1.33 times ≤ SCr increase < 2 times baseline | 1.5 times ≤ SCr increase < 2 times baseline | 1.3 times and 20µmol/L ≤ SCr increase < 1.6 times and 40µmol/L |
|  |  |  |  |
|  |  | or ≥ 0.3 mg/dL (26.5µmol/L) absolute within 48 hours |  |
| Stage 2 | 2 times ≤ SCr increase < 4 times baseline | 2 times ≤ SCr increase < 3 times baseline | 1.6 times and 40µmol/L ≤ SCr increase < 2.2 times and 80µmol/L |
|  |  |  |  |
|  |  |  |  |
| Stage 3 | ≥ 4 times baseline | ≥ 3 times baseline or initiation of RRT | SCr increase ≥ 2.2 times and 80µmol/L |
|  |  |  |  |
|  |  |  |  |
| **Abbreviations:** AKI acute kidney injury; KDIGO Kidney Disease: Improving Global Outcomes; pROCK pediatric reference change value optimized for acute kidney injury; pRIFLE, Pediatric Risk, Injury, Failure, Loss, End-Stage Renal Disease; SCr, serum creatinine; RRT, renal replacement therapy. ^a^Urine output was not used, because records of urine output were not available in the majority of patients. *pRIFLE stage 1=Risk, stage 2=Injury, stage 3=Failure. | | | |

**eTable 3.** Extent of variable-specific missing data among otherwise eligible patients.

| **Data item or status** | **Missing, n** | **Missing, %** | **Available, n** | **Available, %** |
| --- | --- | --- | --- | --- |
| Otherwise eligible cohort after age, SCr-measurement, and CKD exclusions | — | — | 8,611 | 100.0% |
| Outcome status | 42 | 0.5% | 8,569 | 99.5% |
| Age | 0 | 0.0% | 8,611 | 100.0% |
| Sex | 0 | 0.0% | 8,611 | 100.0% |
| ICU unit | 0 | 0.0% | 8,611 | 100.0% |
| Hemoglobin | 702 | 8.2% | 7,909 | 91.8% |
| Glucose | 913 | 10.6% | 7,698 | 89.4% |
| Neutrophil percentage | 785 | 9.1% | 7,826 | 90.9% |
| Platelet count | 618 | 7.2% | 7,993 | 92.8% |
| pCO₂ | 2,041 | 23.7% | 6,570 | 76.3% |
| Chloride | 1,180 | 13.7% | 7,431 | 86.3% |
| Albumin | 1,655 | 19.2% | 6,956 | 80.8% |
| At least one key baseline covariate or outcome status missing | 5,590 | 64.9% | 3,021 | 35.1% |

After excluding patients aged < 28 days (n=1,600), those with fewer than two SCr measurements (n=2,615), and those with chronic kidney disease (n=55), 8,611 patients remained otherwise eligible. Variable-specific missing counts are not mutually exclusive, whereas the number with at least one missing covariate or outcome represents unique patients. Patients with fewer than two SCr measurements were excluded because AKI status could not be determined. Complete-case analysis was used without imputation.

**eTable 4.** Comparison of baseline characteristics between included and excluded patients.

| **Characteristic** | **Included in complete-case analysis (n=3,021)** | **Excluded because of missing data (n=5,590)** | **SMD** |
| --- | --- | --- | --- |
| Age, years | 1.33 [0.42, 4.08] | 0.92 [0.21, 3.12] | 0.170 |
| Sex, n (%) |  |  | 0.039 |
| Male | 1,684 (55.7%) | 3,007 (53.8%) |  |
| Female | 1,337 (44.3%) | 2,583 (46.2%) |  |
| Hemoglobin, g/L | 105 [93, 118] | 108 [95, 121] | 0.120 |
| Glucose, mmol/L | 7.8 [5.9, 10.8] | 7.2 [5.6, 10.0] | 0.101 |
| Neutrophil percentage, % | 68.8 [55.9, 79.4] | 65.1 [51.0, 77.6] | 0.151 |
| Platelet count, ×10⁹/L | 209 [141, 314] | 221 [150, 328] | 0.081 |
| pCO₂, mmHg | 37.4 [32.3, 43.6] | 38.2 [32.7, 45.1] | 0.067 |
| Chloride, mmol/L | 108 [104, 111] | 107 [103, 111] | 0.103 |
| Albumin, g/L | 38.50 [34.70, 41.80] | 39.10 [35.20, 42.30] | 0.089 |
| ICU types, n (%) |  |  | 0.247 |
| CICU | 1,341 (44.4%) | 2,650 (47.4%) |  |
| PICU | 443 (14.7%) | 690 (12.3%) |  |
| SICU | 792 (26.2%) | 1,315 (23.5%) |  |
| General ICU | 445 (14.7%) | 935 (16.7%) |  |

Standardized mean differences (SMDs) were used to assess differences between included and excluded patients; an absolute SMD ≥0.10 was considered indicative of a potentially meaningful imbalance.

**eTable 5.** Paired AUC comparison of Norms-Capped and admission SCr models.

| **AKI criterion** | **AKI form** | **AUC, Admission SCr** | **AUC, Norms-Capped SCr** | **ΔAUC** | **Bootstrap 95% CI** | **P value** |
| --- | --- | --- | --- | --- | --- | --- |
| KDIGO | AKI Stage | 0.785 | 0.803 | 0.018 | 0.005–0.031 | 0.006 |
| KDIGO | Any AKI | 0.784 | 0.802 | 0.019 | 0.006–0.031 | 0.002 |
| KDIGO | Severe AKI | 0.785 | 0.803 | 0.018 | 0.005–0.032 | 0.006 |
| pROCK | AKI Stage | 0.785 | 0.817 | 0.032 | 0.017–0.047 | <0.001 |
| pROCK | Any AKI | 0.783 | 0.798 | 0.015 | 0.003–0.028 | 0.011 |
| pROCK | Severe AKI | 0.785 | 0.817 | 0.031 | 0.017–0.047 | <0.001 |
| pRIFLE | AKI Stage | 0.784 | 0.801 | 0.018 | 0.004–0.031 | 0.009 |
| pRIFLE | Any AKI | 0.783 | 0.797 | 0.014 | 0.003–0.025 | 0.011 |
| pRIFLE | Severe AKI | 0.784 | 0.8 | 0.017 | 0.004–0.030 | 0.013 |

**eTable 6.** Threshold-based classification performance of multivariable logistic regression models for mortality.

| **Baseline SCr method** | **AKI representation** | **Risk threshold** | **Sensitivity (95% CI)** | **Specificity (95% CI)** | **PPV (95% CI)** | **NPV (95% CI)** | **Accuracy (95% CI)** |
| --- | --- | --- | --- | --- | --- | --- | --- |
| AdmSCr & KDIGO | Stage | 0.062 | 0.806 (0.746, 0.857) | 0.621 (0.603, 0.639) | 0.138 (0.119, 0.158) | 0.977 (0.969, 0.983) | 0.634 (0.616, 0.651) |
|  | Any AKI | 0.093 | 0.682 (0.615, 0.745) | 0.739 (0.722, 0.755) | 0.164 (0.140, 0.190) | 0.969 (0.960, 0.976) | 0.735 (0.719, 0.751) |
|  | Severe AKI | 0.062 | 0.810 (0.751, 0.861) | 0.621 (0.603, 0.639) | 0.138 (0.120, 0.159) | 0.978 (0.970, 0.984) | 0.635 (0.617, 0.652) |
| Norms-Capped & KDIGO | Stage | 0.063 | 0.791 (0.730, 0.844) | 0.668 (0.650, 0.685) | 0.152 (0.131, 0.174) | 0.977 (0.969, 0.983) | 0.677 (0.660, 0.693) |
|  | Any AKI | 0.064 | 0.791 (0.730, 0.844) | 0.670 (0.652, 0.687) | 0.153 (0.132, 0.175) | 0.977 (0.969, 0.983) | 0.679 (0.662, 0.695) |
|  | Severe AKI | 0.063 | 0.791 (0.730, 0.844) | 0.667 (0.649, 0.684) | 0.151 (0.131, 0.174) | 0.977 (0.969, 0.983) | 0.676 (0.659, 0.692) |
| AdmSCr & pRIFLE | Stage | 0.069 | 0.777 (0.715, 0.832) | 0.640 (0.622, 0.658) | 0.139 (0.120, 0.161) | 0.975 (0.966, 0.981) | 0.649 (0.632, 0.666) |
|  | Any AKI | 0.070 | 0.777 (0.715, 0.832) | 0.646 (0.628, 0.663) | 0.141 (0.122, 0.163) | 0.975 (0.967, 0.981) | 0.655 (0.637, 0.672) |
|  | Severe AKI | 0.106 | 0.626 (0.557, 0.691) | 0.791 (0.776, 0.806) | 0.184 (0.156, 0.214) | 0.966 (0.957, 0.973) | 0.780 (0.765, 0.795) |
| Norms-Capped & pRIFLE | Stage | 0.079 | 0.725 (0.660, 0.784) | 0.719 (0.702, 0.735) | 0.162 (0.139, 0.187) | 0.972 (0.964, 0.979) | 0.719 (0.703, 0.735) |
|  | Any AKI | 0.091 | 0.692 (0.625, 0.754) | 0.749 (0.732, 0.765) | 0.171 (0.147, 0.198) | 0.970 (0.962, 0.977) | 0.745 (0.729, 0.760) |
|  | Severe AKI | 0.076 | 0.730 (0.665, 0.789) | 0.707 (0.690, 0.724) | 0.157 (0.135, 0.182) | 0.972 (0.964, 0.979) | 0.708 (0.692, 0.725) |
| AdmSCr & pROCK | Stage | 0.067 | 0.796 (0.735, 0.848) | 0.636 (0.617, 0.653) | 0.141 (0.122, 0.162) | 0.976 (0.968, 0.983) | 0.647 (0.629, 0.664) |
|  | Any AKI | 0.069 | 0.782 (0.720, 0.836) | 0.642 (0.624, 0.659) | 0.141 (0.121, 0.162) | 0.975 (0.967, 0.982) | 0.651 (0.634, 0.668) |
|  | Severe AKI | 0.067 | 0.796 (0.735, 0.848) | 0.635 (0.617, 0.653) | 0.141 (0.122, 0.162) | 0.976 (0.968, 0.983) | 0.646 (0.629, 0.664) |
| Norms-Capped &pROCK | Stage | 0.084 | 0.697 (0.630, 0.758) | 0.769 (0.753, 0.785) | 0.185 (0.158, 0.214) | 0.971 (0.963, 0.978) | 0.764 (0.749, 0.779) |
|  | Any AKI | 0.049 | 0.853 (0.798, 0.898) | 0.585 (0.567, 0.604) | 0.134 (0.116, 0.153) | 0.982 (0.974, 0.987) | 0.604 (0.586, 0.622) |
|  | Severe AKI | 0.085 | 0.697 (0.630, 0.758) | 0.772 (0.756, 0.787) | 0.186 (0.160, 0.215) | 0.971 (0.964, 0.978) | 0.766 (0.751, 0.781) |

AKI, acute kidney injury; ICU, intensive care unit; NPV, negative predictive value; PPV, positive predictive value; SCr, serum creatinine.

**eFigure 1.** Diagram of cases included in the study and AKI incidence. AKI was defined and staged according to the KDIGO, pROCK, and pRIFLE definitions. The baseline SCr was calculated based on the AdmSCr or Norms-Capped SCr methods. KDIGO Kidney Disease: Improving Global Outcomes; pROCK pediatric reference change value optimized for acute kidney injury; pRIFLE, Pediatric Risk, Injury, Failure, Loss, End-Stage Renal Disease.

A

B

C

**eFigure 2.** Calibration plots for predicting mortality using different baseline SCr approaches. (**A**) AKI stage; (**B**) any AKI; and (**C**) severe AKI.
